# Diversity and redundancy of the ripening regulatory networks revealed by the fruitENCODE and the new CRISPR/Cas9 *CNR* and *NOR* mutants

**DOI:** 10.1038/s41438-019-0122-x

**Published:** 2019-02-11

**Authors:** Ying Gao, Ning Zhu, Xiaofang Zhu, Meng Wu, Cai-Zhong Jiang, Donald Grierson, Yunbo Luo, Wei Shen, Silin Zhong, Da-Qi Fu, Guiqin Qu

**Affiliations:** 1 Laboratory of Fruit Biology, College of Food Science and Nutritional Engineering, China Agricultural University, 100083 Beijing, China; 2 The State Key Laboratory of Agrobiotechnology, School of Life Sciences, The Chinese University of Hong Kong, Hong Kong, China; 3 Department of Plant Sciences, University of California, Davis, CA 95616 USA; 4 Crops Pathology and Genetics Research Unit, United States Department of Agriculture, Agricultural Research Service, Davis, CA 95616 USA; 5 Plant Sciences Division, School of Biosciences, University of Nottingham, Sutton Bonington Campus, Loughborough, LE12 5RD UK

**Keywords:** Fruiting, Molecular ecology

## Abstract

Tomato is considered as the genetic model for climacteric fruits, in which three major players control the fruit ripening process: ethylene, ripening transcription factors, and DNA methylation. The fruitENCODE project has now shown that there are multiple transcriptional circuits regulating fruit ripening in different species, and H3K27me3, instead of DNA methylation, plays a conserved role in restricting these ripening pathways. In addition, the function of the core tomato ripening transcription factors is now being questioned. We have employed CRISPR/Cas9 genome editing to mutate the *SBP*-*CNR* and *NAC*-*NOR* transcription factors, both of which are considered as master regulators in the current tomato ripening model. These plants only displayed delayed or partial non-ripening phenotypes, distinct from the original mutant plants, which categorically failed to ripen, suggesting that they might be gain-of-function mutants. Besides increased DNA methylation genome-wide, the original mutants also have hyper-H3K27me3 in ripening gene loci such as *ACS2*, *RIN*, and *TDR4*. It is most likely that multiple genetic and epigenetic factors have contributed to their strong non-ripening phenotypes. Hence, we propose that the field should move beyond these linear and two-dimensional models and embrace the fact that important biological processes such as ripening are often regulated by highly redundant network with inputs from multiple levels.

## Fruit ripening: Multiple backup plans

The regulatory circuits that govern the expression of genes required for ripening in tomato plants are highly redundant. Fleshy fruits that use the hormone ethylene to regulate ripening have developed independently multiple times in the history of the angiosperms. Guiqin Qu at China Agricultural University in Beijing and colleagues working on the fruitENCODE project are exploring the genetic and epigenetic basis of this convergent evolution. In tomatoes, three transcription factors have been shown to control ethylene production and regulate ripening. However, when gene editing techniques were used to introduce mutations that interfere with the function of these transcription factors, partial ripening or a delay in ripening was observed. The fact that ripening was not abolished indicates that the ripening process is more robust and complex than previously thought.

## Introduction

For flowering plants, fruits serve as seed dispersal vehicles that have evolved usually from carpels or adjacent floral tissues. Fruit ripening is often described as a developmental process that alters the physiological and biochemical properties of a seed-bearing organ to aid seed dispersal^1^. This enables plants to interact with the coevolving animals that consume the fruits and disperse the defecated seeds to distant locations, and hence increase plant reproductive success^2^. Charles Darwin has also acknowledged its evolutionary advantage as “beauty serves merely as a guide to birds and beasts in order that the fruit may be devoured and the matured seed disseminated”.

However, at the molecular level, transforming an unappealing carpel to a tasty fruit is not a simple task. It requires a complete reprograming of the carpel gene expression network, during which hundreds if not thousands of genes have to be switched on and off in a highly coordinated manner. It is the combined action of these so called “ripening genes” at a precise developmental timing that has transformed nearly every aspect of the carpel tissue, such as color, aroma, flavor, texture, and nutritional content, in order to attract frugivorous as seed dispersers. This process must also be kept under strict regulatory control, as any premature transformation ahead of seed maturation is highly detrimental.

This ripening regulatory network has fascinated scientists for decades, and also has important implications in agriculture worldwide as it affects yield, nutritional value, and shelf life of our horticultural produces. Significant progress has been made using tomato (*Solanum lycopersicum*) as a model to elucidate its genetic and epigenetic basis^3,4^. The tomato ripening model has three key components: the hormone ethylene, ripening transcription factors (CNR, NOR, and NOR), and DNA methylation. However, recent findings from the fruitENCODE project has shown that this model is not universal, as there are at least three different types of transcriptional positive feedback circuits controlling ripening in seven climacteric species^5^. Only tomato has a genome-wide DNA, CG and CHG demethylation, and CHH hypermethylation, while most species used H3K27me3 to regulate the ripening circuits. The H3K27me3 marks and the ripening genes could be traced back to dry and non-climacteric fruits, in which they regulate the floral organ identity and senescence. In addition, gene silencing and genome editing technology have now enabled us to re-examine the tomato fruit ripening transcription factors, resulting in controversial and sometimes conflicting views of its biological functions^6–8^.

In this study, we examined the diversity of the ripening regulatory systems from a genomic and evolutionary perspective. We also provided new evidence showing that CRISPR/Cas9-induced mutations in the core tomato ripening transcription factor *SBP-CNR* and *NAC-NOR* failed to abolish ripening, suggesting that the ripening transcriptional regulatory network is highly robust and has few single points of failure. In a robust system, the lack of mutant phenotypes does not necessarily mean that the gene is not involved in the biological process, while the presence of phenotypes might suggest that the process is not important enough for plants to evolve a backup plan. It is about time for plant biologists to re-evaluate those linear and two-dimensional models generated from traditional genetic studies and often developed solely based on single species studies. After all, complex and important biological processes such as ripening are often regulated by highly redundant transcriptional network with inputs from multiple epigenome levels.

### The tomato ripening model is not universal

The plant hormone ethylene is indispensable for the transition from vegetative growth to ripening in tomato, as well as other climacteric fruits^9,10^. When applied to matured tomato fruits, ethylene can promote ripening, whereas mutants deficient in ethylene biosynthesis or signaling are unable to switch on their ripening process^11–13^. It should be noted that ethylene is unable to trigger ripening in fruits at the immature stage when the seeds are not viable or in other non-fruit tissues. This suggests that a developmental cue is present to coordinate fruit and seed development, and most importantly, prevent premature fruit ripening before seed maturation. Hence, the hypothesis of system 1 and 2 ethylene was often used to describe how ethylene controls fruit ripening^14^. In this model, system 1 ethylene is produced by vegetative tissues at a basal level and is self-inhibitory, while the system 2 ethylene is produced by the ripening fruits and is auto-catalytic.

The genetics behind the system 1 and 2 transition was not fully understood. However, cloning of genes from non-ripening mutants suggested that tomato fruit ripening requires three transcription factors (TFs): MADS-box RIPENING INHIBITOR (RIN), SBP-box COLORLESS NON-RIPENING (CNR), and NAC transcription factor NON-RIPENING (NOR)^11–13^. These three mutants are unable to synthesize the system 2 ethylene, while their system 1 ethylene production, such as wounding ethylene, remained functional. In addition, exogenous ethylene could not restore ripening in these mutants, while system 1 ethylene response such as leaf senescence and seedling triple response are largely unaffected. Therefore, these three TFs were considered to be master regulators of tomato fruit ripening.

**Fig. 1:**
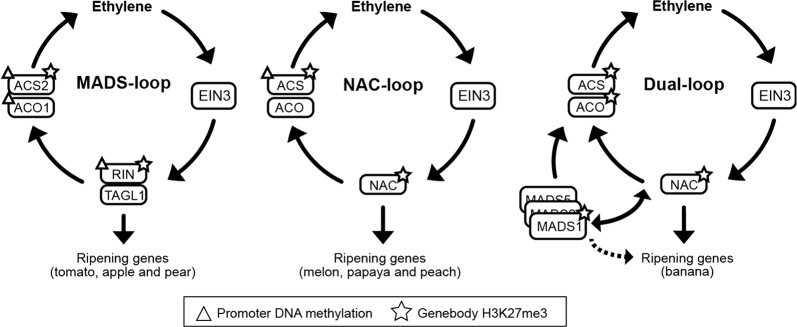
Model for fruit ripening regulation. Ethylene transcription factor EIN3 activates the MADS or NAC TFs, while these TFs activate ethylene biosynthesis genes forming a positive feedback circuit that generates autocatalytic ethylene during ripening. Downstream ripening genes are directly coupled to the loop through these TFs. In leaf and immature fruits, the loop is repressed with key genes associated with DNA hypermethylation in the promoter or repressive histone mark H3K27me3 in the gene body

Among these three ripening TFs, RIN is the best studied. Extensive ChIP-Seq experiments have shown that it could directly bind to the promoter of tomato ripening genes, including cell wall softening genes *PG*, *EXP1*, *CEL2*, aroma biosynthesis genes *LoxC*, pigment formation genes *PSY1*, and additional TF genes, such as *NOR*, *CNR*, *AP2a*, *FUL1/2*^4,5,15^. Most importantly, RIN also binds to the promoter of ethylene biosynthesis genes *ACS2* and *ACO1*^5,16^. Promoter motif analysis and ChIP-Seq also confirmed that ethylene transcription factor EIN3 binds to the promoter of *RIN*, which completes a positive feedback loop that fits the autocatalytic system 2 ethylene proposed by McMurchie et al., 1972^14^. With this MADS-loop activated in ripening fruit, a small amount of ethylene could rapidly amplify itself and drives the downstream ripening gene expression (Fig. 1). However, ethylene is a stress hormone, and continued ethylene synthesis could disrupt plant growth and even cause tissue death such as leaf senescence and flower abscission^10,17^. Hence, it is vital for plants to repress this positive feedback loop in non-ripening tissues. DNA methylation was thought to be responsible for repressing tomato ripening gene^4^. To our surprise, we did not find conserved DNA methylation change in other species’ ripening gene promoters^5^, suggesting that it might be a unique event for tomato.

H3K27me3 is associated with silencing of key developmental genes in both animals^18^, and is catalyzed and bound by the Polycomb repressive complexes, which could condense chromatin and silence gene expression. In plants, H3K27me3 is best known for silencing the flowering regulator *FLOWERING LOCUS C* and floral homeotic gene *AGAMOUS*, both of which are MADS-box transcription factors^19^. It turns out that H3K27me3 played a conserved role in restricting the expression of climacteric fruit ripening genes and even their orthologs in both dry fruits and ethylene-independent fleshy fruits (Fig. 1). This suggests that fruit ripening mechanisms originated from preexisting pathways in the ancestral angiosperms. In addition, plants like tomato, peach, and banana have not just borrowed the genes from their ancestors to construct the ripening circuits, but also their epigenetic marks.

### Reexamine the ripening master regulators CNR, RIN, and NOR

Much of what we know about the ripening regulation came from the three tomato TF mutants (*cnr*, *rin*, and *nor*) that failed to ripen. The *rin* mutant is caused by a DNA deletion, resulting in a truncated *RIN* fused to an adjacent MADS gene *MACROCALYX*^12^. It was once thought that the chimeric RIN-MC fusion protein could not have biological functional, and *rin* is a loss-of-function mutant, while recent evidence suggests otherwise. CRISPR/Cas9 knockout and RNAi silencing of RIN in the wild-type tomato only recreated a partial non-ripening phenotype distinct from the complete lack of ripening in the *rin* mutant^5,6^. On the other hand, knockout or RNAi silencing of the chimeric *RIN-MC* mutant protein in *rin* background could partially restore ripening. These reults suggest that *rin* is in fact a gain-of-function mutant^8^.

**Fig. 2:**
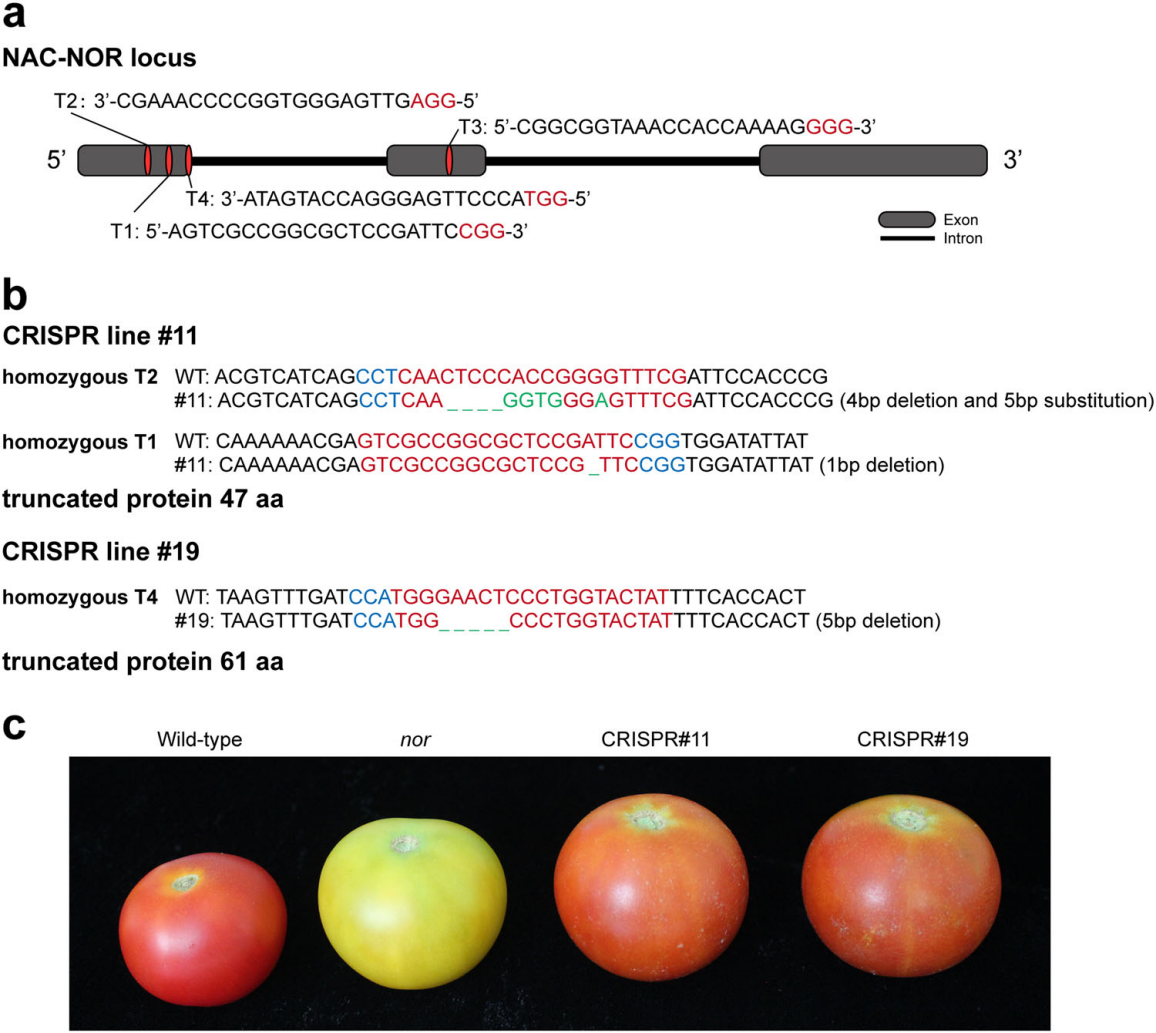
Partial non-ripening phenotype of NOR CRISPR/Cas9 knockout. **a** Position of the NOR gRNA target sites (T2 231–209 bp, T1 281–302 bp, T4 363–341 bp, T3 1169–1191 bp). **b** Sanger sequencing of the CRISPR edited sites in line #11 (four bases of CTCC located in 215–218 bp and one base of A located in the 269 bp were deleted, CACCGGG located in 219–225 bp were substituted to GGTGGGA) and #19 (GAACT which were located in 347–351 bp were deleted). Red letters indicate the gRNA target sites, green letters represent edited sites and blue letters represent the protospacer adjacent motif (PAM). **c** The partial non-ripening phenotype of CRISPR/Cas9 fruits compared with that of the wild-type, and non-ripening *nor* mutant

**Fig. 3:**
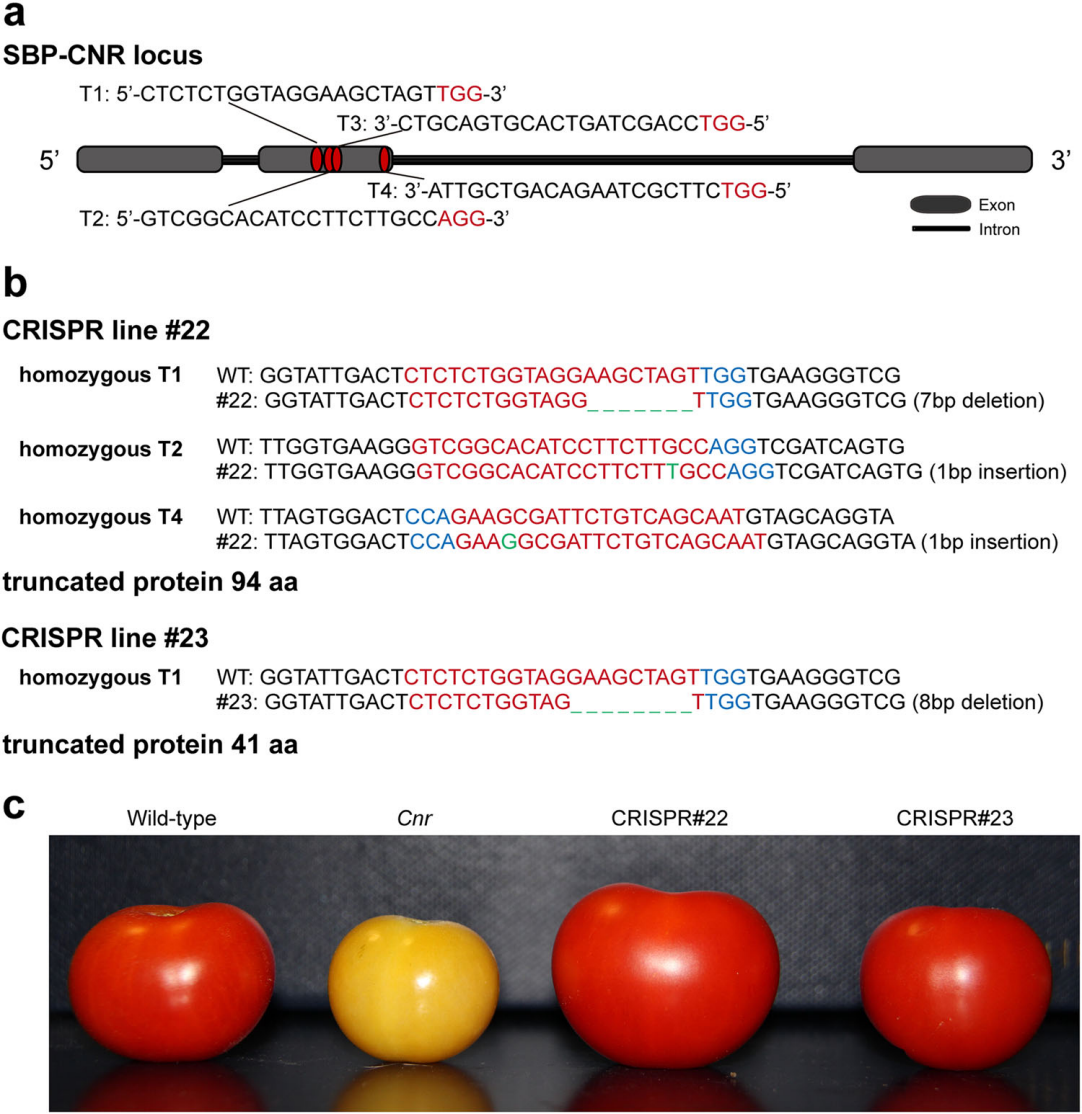
Phenotype of the CNR CRISPR/Cas9 knockout. **a** Schematic illustration of four gRNA target sites in CNR locus (T1 535–557 bp, T2 564–586 bp, T3 604–582 bp, T4 712–690 bp). **b** Sanger sequencing of the CRISPR edited sites in line #22 (seven bases of AAGCTAG located in 547–553 bp were deleted, one base of T and one base of G were inserted after 580 bp and 695 bp, respectively) and #23 (eight bases of GAAGCTAG located in 546–553 bp were deleted). Red letters indicate the target sites, green letters represent edited sites, blue letters represent the PAM. **c** The CNR CRISPR/Cas9 fruits only showed 2–3 days of delayed ripening compared with that of the WT and the fruits obtained the full color finally, while the original *cnr* mutant failed to ripen

To examine the remaining *CNR* and *NOR* genes, which were also believed to function as master regulators necessary for ripening, we have used CRISPR/Cas9 to generate multiple potential true knockout mutations in their gene loci. We found that the *CNR* CRISPR lines only showed a delayed ripening phenotype, while the *NOR* lines showed partial non-ripening phenotypes similar to the RIN CRISPR/Cas9 mutants. Both are different from the strong non-ripening phenotypes of their natural mutants (Figs. 2 and 3).

**Fig. 4:**
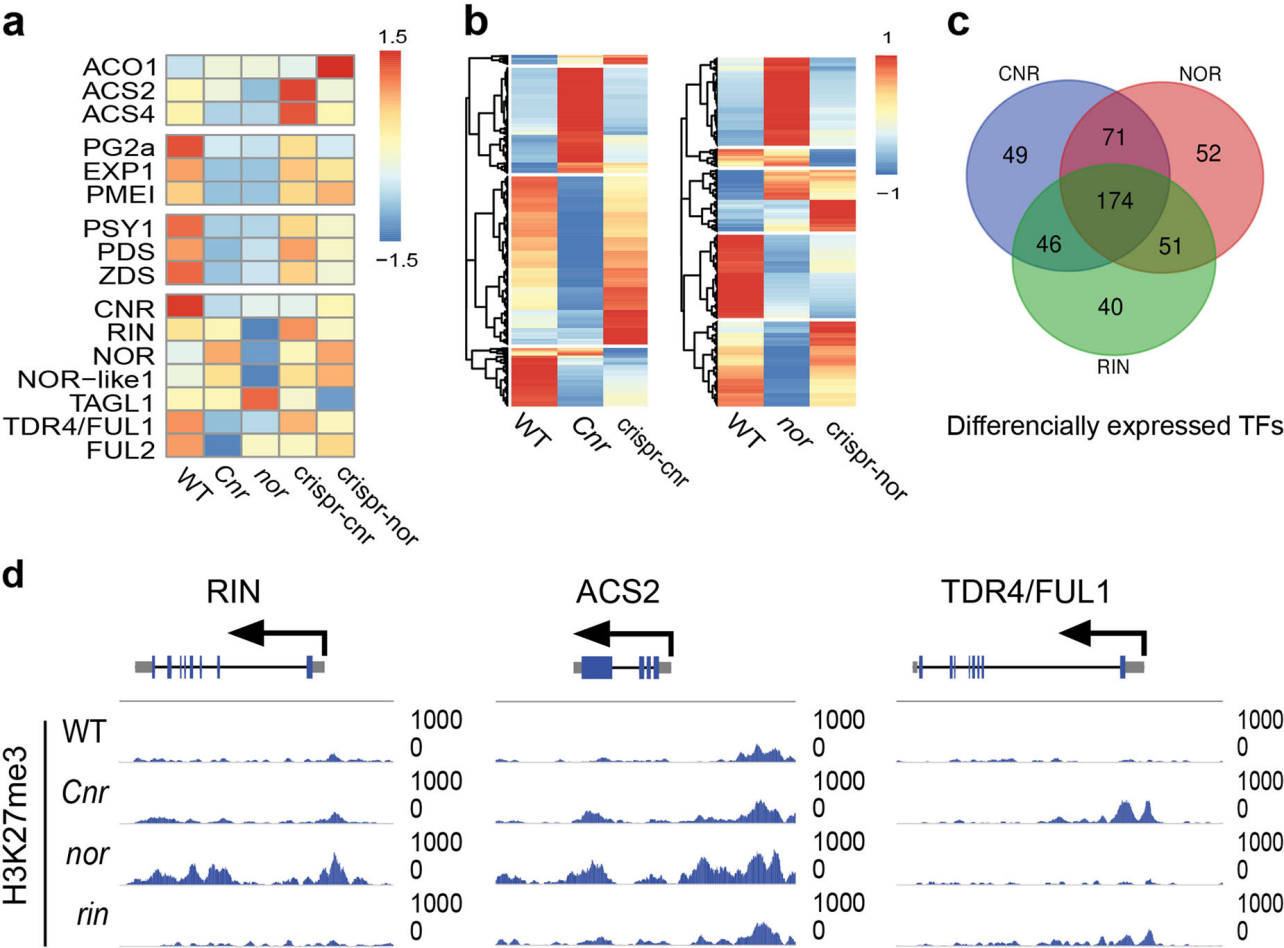
Gene expression and H3K27me3 changes in the mutants. **a**-**b**, Heatmap showing key ripening gene expression in the wild-type, CRISPR/Cas9 lines and original mutants. **c** Venn diagram showing differentially expressed transcription factor genes in the CRISPR/Cas9 lines and original mutants when compared to wild-type fruit. RNA-Seq data from the RIN RNAi silencing were used to identify differentially expressed genes in *rin*. **d** Altered histone modification H3K27me3 in the original *Cnr*, *rin*, and *nor* mutants

RNA-Seq analysis of their fruits showed that the expression of the hallmark ripening genes such as *ACS2, ACO1, PSY, PG*, and *EXP* are not fully silent in the CRISPR/Cas9 lines, as compared to the natural mutants (Fig. 4a, b and Table S1). In the original *nor* mutant, the *ACS2* and *RIN* genes loci are associated with hyper-H3K27me3 marks, while in the *cnr* epi-mutant, hyper-H3K27me3 is also found in ripening transcription factor *TDR4/FUL1* (Fig. 4c), suggesting that H3K27me3 could play a role in their strong non-ripening phenotypes.

The original *cnr* epi-mutant was thought to be caused by hypermethylation in a 286-bp region of the *SBP-CNR* gene promoter, which reduced its gene expression^13^. Genome-wide DNA methylation analysis has shown that the cnr fruit experienced a whole-genome DNA hypermethylation^4,5^. VIGS silencing of the DNA methylase *SlCMT3* can restore ripening in the *cnr* fruit^20^, suggesting that besides the involvement of the SBP-CNR itself, DNA hypermethylation in other loci might have contributed to its non-ripening phenotype, or there are unknown gain-of-function properties in the *cnr* mutant locus.

### How to define transcription factor function in the post genomic era

Transcription factor (TF) are sequence-specific DNA binding protein capable of transcription regulation. One of the most important attributes of a TF is the sequence preference, the binding sites to be precise. Therefore, the *cis-*regulatory elements in the promoter of downstream target genes, not just the biochemical properties of the TF protein itself, largely define the biological function of a TF. Unlike a common enzyme coding gene, we must consider the function of a TF from two levels: the biochemical function of the TF protein itself and the regulatory function that is determined by its target genes’ *cis*-regulatory elements. For example, when an apple TF could rescue a tomato TF loss-of-function mutant, it does not necessarily mean that these two TFs have conserved function, because they might regulate a completely different set of genes in their native genome environment.

This is further complicated by the fact that TFs also need to signal other proteins like the Mediator and RNA polymerase II to initiate transcription or recruit chromatin modifier to indirectly activate or repressive transcription. Hence, the co-factors also influence the TF’s biochemical properties, not to mention that a TF could lack effector function on its own, and instead act by steric mechanisms. It is likely the case for the original *NAC-NOR* and *MADS-RIN* mutants, where the mutant protein is either truncated or fused to others, that they retained their putative DNA binding domains. Although they might lack transcriptional activation function, they could block other TFs from binding to the same sites, leading to a gain-of-function non-ripening phenotype.

### TF-gene regulatory network is often complex and redundant

The technologies for studying TF have also evolved. First developed for mapping in vivo binding sites of human TFs genome-wide, ChIP-Seq represents a dramatic improvement compared to other analysis methods such as EMSA, ChIP-PCR, and ChIPchip^21^. However, the unparalleled resolution of ChIP-Seq presents a new challenge for the study of TF function, and misinterpretation of the ChIP-Seq data often created more problem than it solved. The most common mistake is to define potential TF targets based on the present of ChIP-Seq binding, and then only cherry-pick the genes with altered expression when the TF is knocked out or over-expressed. We should learn from the mammalian research, where hundreds of TF ChIP-Seq experiments have been performed to map binding sites of all highly expressed TFs in different cells and tissues^22,23^. These large-scale experiments showed that TF could bind to tens of thousands of sites and each gene is targeted by multiple TFs. Although TF binding is strongly associated with gene transcription, knockdown of specific TF could only impact a small number of its targets, meaning that the regulatory network is highly redundant.

In the tomato genome (annotation version ITAG2.4), there are 2026 tomato protein coding genes with putative DNA binding domain(s) that might be considered as transcription factors, and 516 of them are expressed (RPKM > 10) in the ripening tomato fruit^4,24^. Among those, 25 are NAC-class, 16 are MADS-class, and 7 are SBP-class TFs (Table S1). Over 400 TF genes are differentially expressed in the *Cnr*, *rin*, and nor mutant and their CRISPR/Cas9 or RNAi lines (Fig. 4), suggesting that the ripening TF regulatory network is also complex and robust.

**Fig. 5:**
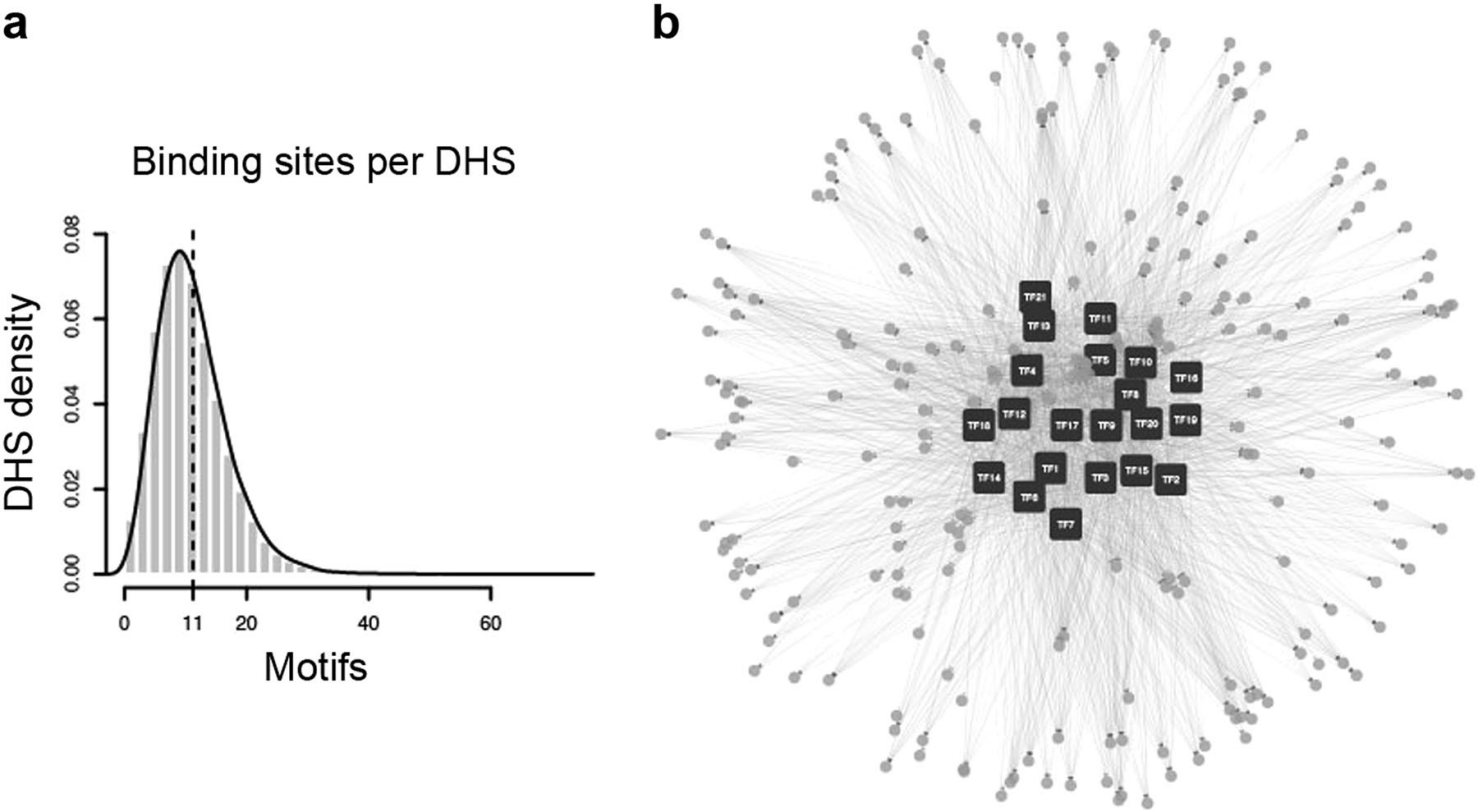
Complexity and redundancy of the tomato ripening transcription regulatory network. **a** Distribution of potential TF binding motifs in all tomato open chromatin regions. The mean is indicated by the vertical dash line. **b** A schematic diagram showing the redundancy of plant transcription network using 20 maize transcription factors and 500 target genes from the C3C4ENCODE project

Although the fruitENCODE project has not yet generated a comprehensive TF ChIP-Seq dataset comparable to those of the mammalian ENCODE projects, we could still estimate the complexity of this TF network using the gene expression data and TF binding motifs in the promoter open chromatin. As the precise location of promoter open chromatin regions (DNaseI hypersensitive site, DHS) for nearly all tomato genes have been now defined by DNaseI-Seq^5^, we have counted the potential TF binding sites in each DHS regions using known plant TF motifs from JASPAR and TRANSFAC database (Fig. 5a). On average, each tomato DHS has 11 potential TF binding sites, suggesting that ripening genes are targeted by multiple TFs. Therefore, the actual tomato transcriptional regulatory network is most likely to mimic the mammal ones, where TFs themselves are heavily interconnected and genes are targeted by highly redundant TFs (Fig. 5b). We should not be surprised to see that these three master ripening regulators were not “required” for ripening, as there might not be a singular master regulator in any highly redundant biological network.

**Evolution of ripening is a transcription reprogram in the carpel tissue**

Ripening fleshy fruit is a product of convergent evolution. It is difficult to fully understand ripening regulation if we do not look beyond the tomato model. We have compared the climacteric fruit ripening genes with their orthologues in dry and non-climacteric fruits. It turns out that all these transcription factors, hormone signaling genes, ripening genes, and epigenetic factors are already present in the non-climacteric and dry fruit species^5^. Hence, the plants did not invent new genes to transform the carpel into a fleshy fruit. Instead, they just rewired their gene regulatory network to express existing genes in the carpel tissue. With the exception, however, that genome or gene duplication could create additional genetic materials for plants without the need for repurposing the old ones.

We propose that perhaps the first step of evolving a climacteric fruit is to re-direct the ethylene signaling pathway to regulate “ripening genes” in the carpel tissue, which should be a logical choice as ethylene is well known to be synthesized during carpel senescence. It also has the advantage of being a gas signal molecule that could diffuse from cell to cell without the need of a transporter. In addition, there has been evidence suggesting that even the dry fruited *Arabidopsis* can produce a respiration burst during carpel senescence^25^. Most intriguingly, it is also ethylene dependent and requires a NAC transcription factor AtNAP1, which is a homolog of the tomato NOR, as well as other species’ NAC TFs involved in the ripening positive feedback loops.

After committing the hormone ethylene to regulate ripening, plants still have to assign TFs to control the ripening genes. Although it sounds easy, it actually requires gaining *cis-*regulatory elements in the promoter open chromatin regions of hundreds or thousands of genes. Therefore, these ancestral plants faced a difficult choice regarding the TF and its regulatory network topology. The shortest route might be to directly couple the “ripening genes” to the ethylene signaling pathway. There are conserved TFs controlled by ethylene, such as EIN3 and ERF class TFs. However, if the plants want to restrict ripening to matured fruit tissues, genes directly regulated by these ethylene TFs must be repressed in other tissues, where ethylene signal could be accidentally switch on by biotic or abiotic stresses. Evolving new epigenetic regulations such as methylation or H3K27me3 to repress thousands of ripening genes could be costly. The fruitENCODE project showed that all 7 climacteric fruits took a longer route, which is to create a “new” TF controlled by ethylene, and then couple the ripening genes to it. By taking this longer route, plants only need to control a single or a few TF genes to achieve a ripening fruit-specific gene expression program.

The three ethylene positive feedback circuits discovered so far are perhaps the most optimal designs that have survived the process of natural selection (Fig. 1). However, since the fruitENCODE project could only examined 7 climacteric species with high quality reference genome assemblies, future study of additional plants, as well as wild species of the domesticated crops might shed light on much more diverse ripening circuits.

### Misinterpretation of big data often leads to the wrong conclusion, a lesson from the genome-wide DNA methylation analysis

Next generation sequencing technologies have enabled genome-wide analysis of biological processes at multiple dimensions. While addressing the same underlying questions, these big data has different properties that requires a complete overhaul of the data analysis and interpretation methods. For example, DNA methylation is often considered as a repressive epigenetic mark. This concept originated from observation that high DNA methylation is associated with inactive transcription. The transposable elements in the genome are heavily methylated, while global demethylation in endosperm tissues are associated with their transcriptional reactivation^26,27^.

Silencing the tomato DNA demethylase using RNAi can prevent fruit ripening, and it has been later confirmed by CRISPR/Cas9 mutation^28,29^. This suggests that DNA methylation is involved in ripening control. However, we still do not know which gene(s) is directly controlled by DNA methylation. There are over ten thousand DNA demethylation sites (hypo-DMRs) identified in the ripen vs. immature tomato fruit pairwise comparison^4^. The DMRs are enriched at the 5′ end of genes, and many of them are associated with transcription factor RIN binding sites. This result is robust to the choice of the distance used to define the putative promoter region and TF binding regions, as well as different statistical models and thresholds used to define DMR. Hence, it is tempting to suggest that DNA methylation in the promoter region could affect TF binding leading to repression of ripening genes.

However, it is often overlooked that there are many genes whose expression profiles are not associated well with DNA methylation changes. For example, some key ripening genes such as *ACS2* and *CNR* are already expressed in the developing tomato fruit before their promoter regions are demethylated. In addition, there are many down-regulated genes associated with promoter demethylation during tomato fruit ripening^4,5,29^. Again, we could conveniently expand the function of DNA methylation and suggests that it can activate transcription. This is indeed supported by recent experimental evidence^30^. However, it is meaningless to discuss DNA methylation change on its own as it affects thousands of genes either directly or indirectly, and can both activate and repress transcription.

In addition, if we knockout key tomato DNA methylase genes, which will surely affect the expression of thousands of genes and perhaps also disrupt many biological processes such as ripening, should we start to claim that DNA hypermethylation, not just demethylation, also regulate fruit ripening? The same logical fallacy could be applied to any “global” factors, such as protein post-translational modification and small RNA biogenesis, or even transcription itself, as we can easily disrupt any complex developmental processes by altering these core machineries. But what is the significance of claiming that these core machinery is necessary for ripening? The key question is not whether global regulators such as DNA methylation are involved in ripening. Instead, we should ask which gene(s) necessary for ripening is directly regulated by DNA methylation.

This might be new to the horticulture field, but it has been well recognized in the mammalian research. The human ENCODE project has systematically profiled gene expression, DNA methylation, and chromatin accessibility in multiple cell lines and tissues^31^. They found a strong anti-correlation between DNA methylation and chromatin accessibility. Hence, it was proposed that the majority of the DNA methylation changes are passive and only a small number of them could be actively controlling TF binding. DNA methylation is likely to be automatically deposited onto DNA when a TF vacates the binding sites and the promoter region become inaccessible.

In the fruitENCODE project, we did not observe conserved DNA methylation changes targeting the ripening genes in different plant species, neither did other fruits experience a tomato-like whole-genome demethylation. In fact, all of them have local DNA methylation changes, and we found the same inverse-correlation between their methylation change and chromatin accessibility change^5^. These observations suggest that the majority of the promoter DNA methylation changes in fruits are passive marks of promoter chromatin remodeling, just like the mammalian ones. Hence, it is futile and often misleading to discuss the function of DNA methylation when it is taken out of the context of the chromatin environment.

## Concluding remarks

Fleshy fruit is a classic example of convergent evolution and as they have developed independently multiple times in the history of the angiosperm, this already suggests that they could have evolved different mechanisms to achieve this. However, some of them, mainly those classified as climacteric fruits, utilize the same plant hormone ethylene to regulate ripening. Their ripening is associated with a burst of respiration, and most importantly, autocatalytic ethylene synthesis, which is necessary for ripening to initiate and progress. In all seven cases (tomato, apple, pear, melon, papaya, peach, and banana) that have been examined in the fruitENCODE project, the autocatalytic ethylene is produced by positive feedback loops formed between ethylene and the so-called “ripening TFs” (Fig. 1). Many genes required for ripening are coupled directly to this autocatalytic feedback loop through these TFs, which are controlled by conserved tissue-specific H3K27me3 marks^5^. We propose that evolving this strategy enable plants to control most if not all ripening genes through a few key TFs, which is efficient and could be of significant evolutionary advantage.

Interestingly, the lack of more diverse ripening circuits appears to suggest that the evolution of fruit ripening was constrained by some unknown factors, which could be the limited optimal signaling molecules and genetic and epigenetic materials in the carpel tissue. What we have observed might be the most optimized path(s) for plants to evolve a ripening fruit, which is to repurpose an existing carpel senescence gene, or use the duplicated floral organ identify genes^5^. However, further investigations in more species are necessary in order to show whether other regulatory systems do exist.

Another important lesson from the fruitENCODE project and these CRISPR/Cas9 mutants is that ripening is controlled by a complex and highly redundant transcriptional regulatory network. Many biological processes are robust to change, and such redundant system can tolerate multiple perturbations. The evolutionary benefit of a robust system is the increase of neutral mutations, meaning the population could carry more genetic variations that might be useful in the future. Analysis of such a complex system at a single level could easily lead to an incomplete picture such as the traditional genetic approaches or examining a single epigenetic factor while ignoring others. Therefore, if we want to fully understand a complex plant developmental process such as fruit ripening, we might need to adopt a more holistic approach considering regulatory inputs from multiple dimensions such as 3D chromatin organization, DNA methylation, chromatin accessibility, histone modification, TF network and transcriptome. How to combine the different advantages of traditional genetic experiment and the big data driven approaches is a new challenge for plant biologists.

## Materials and methods

### Plant materials

Wild-type (WT) tomato plants (*Solanum lycopersicum* cv. Ailsa Craig), *nor* and *Cnr* mutants, and transgenic lines were grown in a greenhouse under controlled condition with natural light. Fruits samples of WT, *nor* and *Cnr* mutants, and transgenic lines were harvested at the red-ripe stage. Pericarp tissues were collected, immediately frozen in liquid nitrogen and stored at −80 °C for RNA isolation.

### CRISPR/Cas9 knock out of *NOR* and *CNR*

CRISPR-P (http://cbi.hzau.edu.cn/crispr/) was used to select four specific sgRNAs that targeted the tomato *NOR* or *CNR*, respectively (Figs. 2 and 3). The gRNAs were amplified and cloned into the pYLCRISPR/Cas9Pubi-H binary vector using the Golden Gate method. The resulting pYLCRISPR/Cas9Pubi-H-SlNOR and pYLCRISPR/Cas9Pubi-H-SlCNR vectors were transformed into the wild-type tomato Ailsa Craig using stable *Agrobacterium tumefaciens*. Genomic DNA was isolated from young leaves of transgenic lines and PCR amplified using primers flanking the target sites. The PCR products were sequenced to identify mutations.

### RNA-Seq and data analysis

Total RNA was extracted from the pericarp tissues of WT, *nor* mutant, NOR CRISPR line #11, *Cnr* mutant, CNR CRISPR line #23 fruits at the red ripe stage using the RNeasy Mini Kit (Qiagen, Germany). Three biological replicates were used for TrueSeq library preparation. Illumina sequencing was performed on HiSeq2500 using the PE150 mode. The raw data were mapped to the tomato reference genome v3 using tophat, and differentially expressed genes were called using DEGseq2.

## Supplementary information

Dataset 1
